# Assessment of disability of leprosy in Bangladesh: A hospital-based study

**DOI:** 10.1371/journal.pone.0357047

**Published:** 2026-08-27

**Authors:** Hafez Mohammad Nazmul Ahsan, Shaheera Rahman, Labida Islam, Monjur Rahman, Farhana Zaman, Rowshan Ara, Hossain Mohammed Omar Khayum, Saidur Rahman Mashreky

**Affiliations:** 1 Shaheed Suhrawardy Medical College, Dhaka, Bangladesh; 2 BRAC University, Dhaka, Bangladesh; 3 Orbis International, Dhaka, Bangladesh; 4 North South University, Dhaka, Bangladesh; 5 Universal Medical College, Dhaka, Bangladesh; Bai Jerbai Wadia Hospital for Children, INDIA

## Abstract

**Background:**

In Bangladesh, Leprosy remains a key challenge in the public health sector, especially in the rural areas where detection of the disease in the early stages and access to healthcare are still limited. Even though there have been national efforts to eliminate the disease, the burden of the disease remains high. There are people who face consequences as a result of the disease. The aim of this study is to understand the extent of the burden of leprosy among patients and also identify the clinical and socio-demographic factors that are associated so that intervention strategies can be formed.

**Methods:**

A hospital based cross-sectional study was carried out in the two centers for leprosy treatment from March 2024 to May 2024. Face to face interviews were conducted to collect data from 176 patients who were diagnosed with leprosy. Furthermore, standardized assessment tools such as the World Health Organization (WHO) disability grading system, the Patient Health Questionnaire-9 (PHQ-9) for depressive symptoms, the Screening of Activity Limitation and Safety Awareness (SALSA) scale for activity limitation, and a social discrimination questionnaire were also used. Data were analyzed in Statistical Package for the Social Sciences (SPSS) version 25 using descriptive statistics, chi-square and Fisher’s exact tests, and multivariable logistic regression. A p-value below 0.05 was considered statistically significant.

**Results:**

Among the 176 participants recruited at specialist leprosy centers, 72.7% had Grade 2 impairment and 94% had multibacillary leprosy; these proportions describe a facility-based sample and are not national prevalence estimates. Most common were disabilities such as claw hands, drop feet and ocular complications. Grade 2 impairment was strongly associated with the type of leprosy (Fisher’s exact p < 0.001) but did not differ significantly by sex (p = 0.21), although the pattern of individual impairments did vary by sex. In multivariable logistic regression, multibacillary classification was the strongest independent predictor of Grade 2 impairment (adjusted OR 15.70, 95% CI 1.94–126.75). A total of 67.6% (119/176) screened positive for at least mild depressive symptoms on the PHQ-9 (score ≥5); the PHQ-9 is a screening instrument and these proportions do not represent clinically diagnosed depressive disorder. Depressive symptoms increased with impairment grade (p < 0.001), and women scored higher than men (mean 8.72 versus 6.53, p = 0.002). Social discrimination was also experienced by the patients and it was seen that 30.1% did not attend school (2.4% forced to leave), 8% were not married (28.6% unable to marry, 8.6% separated), 24.1% were forced to leave jobs, 20.4% had promotions affected, 12.8% were refused employment, 10.3% were restricted in jobs which impacted their economic status and mental health.

**Conclusions:**

Among people affected by leprosy attending specialist centers in Bangladesh, the burden of severe impairment, depressive symptoms and social discrimination was substantial and was concentrated among those with multibacillary disease and longer delays before treatment. Because recruitment was facility-based and by convenience sampling, these proportions are likely to overstate the burden in the wider leprosy population and should not be read as national estimates. The findings nonetheless support strengthening early detection, rehabilitation and mental health provision for people affected by leprosy.

## Background

Leprosy, also known as Hansen’s disease, is a chronic infectious disease which is caused by the infection of *Mycobacterium leprae* and *Mycobacterium lepromatosis* [1]. It mainly affects the skin, peripheral nerves, the mucosa of the upper respiratory tract and the eyes [2,3]. Leprosy was declared eliminated by WHO in the 2000 and was not considered a public health threat, however, around 200,000 new cases were reportedly all over the world in 2017 which again made leprosy a major public health concern [4,5]. In addition to the physical disabilities, people affected by leprosy, along with their family members and caregivers are also faced with discrimination from society [6]. Nonetheless, leprosy is a curable disease and if it is detected in the early stages with access to proper treatment, it is possible that there would be significant reduction in disability in the eyes, hands, and feet [1].

Over the previous years, the number of reported cases of leprosy in America has fluctuated. In 2018, the reported cases of leprosy were 30,957 which reduced in 2019 and 2020–19,195 cases, however, ever since, the number of cases of leprosy has seen an upward trend with 24,773 cases in 2023 [7]. In 2023, globally, there were 182,815 new cases of leprosy [7]. The prevalence of leprosy is highest in the developing countries of south-east Asia, accounting for 70% of the cases worldwide according to the records from 2013 with India, Brazil and Indonesia being the three countries that have the highest annual cases of leprosy [8]. As per data from 2023, it was seen that 12 other countries including Bangladesh continue to report somewhere between 1000−10,000 new cases [3]. Even though multidrug therapy (MDT) is available for the treatment of leprosy, it still remains a major challenge in low and middle income countries where localized endemicity exists due to a delay in seeking treatment of lack of health education or hygiene or because the dependency of the patients on non-medical methods such as herbal treatments or traditional healers [9]. The disease disproportionately affects marginalized communities, causing an increase in the socioeconomic disparities and hindering access to healthcare services [10].

Bangladesh had made significant strides in leprosy control, achieving the WHO’s elimination target at the national level in 1998, however, it is still endemic in some districts of Bangladesh [11]. Physical impairments associated with leprosy, including limb deformities, ulcerations, and ocular complications, are major contributors to disability [12]. These impairments not only impact individual mobility and daily functioning but also lead to unemployment, social exclusion, and mental health disorders such as depression and anxiety [13]. Psychosocial challenges of the affected individuals are also intensified by stigma and misconceptions about leprosy [14].

It is important to determine the level and the effects of disabilities among the patients with leprosy to develop effective intervention strategies [15]. The previous research has recorded the significance of early detection and rehabilitation services to reduce the occurrence of long-term complications in patients with leprosy [16]. Nonetheless, little comprehensive information was found on the burden of disability among people affected by leprosy in Bangladesh. Knowledge of socio-demographic, clinical, and psychosocial determinants of disability may be used to formulate specific health policies and enhance the support systems of patients [17].

The research aims to examine the level of existing disability and its intensity in patients with leprosy in Bangladesh using a hospital-based methodology. Through the assessment of the relationships between the disability grades and the socio-demographic factors, the activity limitations, and mental health status, this study aims to contribute to the understanding of the overarching effects of the leprosy-related impairments. The results can be used to make evidence-based recommendations on healthcare interventions, rehabilitative programs, and policy programs that can address the burden of disabilities associated with leprosy in Bangladesh.

## Methodology

### Study design

The study utilized a cross-sectional study design. The study was conducted at Filaria, Thalassemia and General Hospital, Savar; Institute of Leprosy Control Hospital, Mohakhali; Government Leprosy Hospital, and Danish Leprosy Mission, Nilphamari; and Filaria Hospital & CDC, Saidpur. Since January 2024, the planning and preparation of the study had started. The ethical clearance was requested in January 2024 which was received in February 2024 after which the data collection process started from 03/03/2024 and continued till 30/05/2024.

### Study population

Both male and female patients with leprosy of all ages who were willing to participate in the study were included however, the patients who were not willing to participate in the study were excluded.

### Study sampling

The initial sample size estimated was 371 which was calculated using the Cochran’s formula.

The formula used was:

n = sample size

z = z-score for the desired confidence level (1.96 for 95% confidence)

p = anticipated prevalence of disability (32%)

d = desired margin of error


n= 1.96^2*.32(.68)/.05^2

$$\begin{array}{c} \text{n}=\text{\textasciitilde{}334}.\text{372864 }\sim \text{\textasciitilde{}334} \end{array}$$

The target sample came to be 371 after an attrition rate of 10% was adjusted. However, because of limited patient availability during the data-collection window and a fixed study timeline, data were collected from 176 participants using convenience sampling. The achieved sample estimates a prevalence of approximately 32% to within ±6.9% at 95% confidence, compared with ±4.7% for the planned sample of 371; the study is therefore less precise than originally intended, and this is considered further in the Limitations.

### Data collection

Face-to-face interviews were carried out where a semi-structured questionnaire was used to assess the seriousness and prevalence of disabilities among patients with leprosy. Depression for patients with leprosy was assessed using the Patient Health Questionnaire-9 (PHQ-9) and the social stigma was assessed using the Discrimination assessment form. Before initiation of data collection, pre-testing of the questionnaire was done. Two days of extensive training of the data collectors was conducted by researchers. One field manager and ten data collectors were recruited for the data collection process. Data quality was monitored by the researchers at specific time interval.

### Data processing and analysis

Basic demographic data including age, gender, marital status, employment status was obtained for leprosy. Disability grading was done according to the WHO Operational Guidelines for the Enhanced Global Strategy 2011–2015 (Paucibacillary and Multibacillary).

During the initial stages, the quantitative data was entered in Microsoft Excel for logical checking. Records with incomplete data were excluded during logical checking, and only valid and complete records were transferred into SPSS version 25 for final analysis. A questionnaire was considered incomplete if the WHO disability grade, more than two PHQ-9 items, or any core socio-demographic variable was missing. No imputation was performed; all analyses are complete-case. Analysis proceeded in two phases. Descriptive statistics comprised means, medians, standard deviations and percentages. Inferential analysis used the chi-square test for associations between categorical variables, with Fisher’s exact test substituted wherever any expected cell count fell below five. A p-value below 0.05 was considered statistically significant, and all tests were two-tailed.

Depressive symptoms were assessed with the PHQ-9, administered in Bengali by trained interviewers. Each of the nine items was scored from 0 to 3 according to symptom frequency over the preceding two weeks, giving a total score between 0 and 27. Severity was categorized as minimal (0-4), mild (5-9), moderate (10-14), moderately severe (15-19) and severe (20-27). A score of 5 or above was treated as screening positive for at least mild depressive symptoms, and a score of 10 or above as moderate or worse. The PHQ-9 is a screening instrument and does not establish a clinical diagnosis.

Activity limitation was assessed with the SALSA scale, which yields a summed score across items covering mobility, self-care, work and dexterity. Total scores range from 10 to 80, with higher scores indicating greater limitation, and were categorized as no significant limitation (10-24), mild (25-39), moderate (40-49), severe (50-59) and extreme limitation (60-80).

Because Table 3 reports eleven simultaneous comparisons, unadjusted p-values are presented alongside p-values adjusted by the Benjamini–Hochberg false discovery rate procedure, and these comparisons are interpreted as exploratory.

Multivariable logistic regression was used to identify factors independently associated with Grade 2 disability, with age, sex, leprosy classification, delay between symptom onset and first consultation, treatment duration and residence entered as candidate predictors. Adjusted odds ratios are presented with 95% confidence intervals (CI). A second model examined moderate or worse depressive symptoms (PHQ-9 ≥ 10) as the outcome, adjusting for age, sex, disability grade and experience of discrimination. Model fit was assessed by the Hosmer-Lemeshow test, the McFadden pseudo R-squared and the area under the ROC curve. Because only 11 participants had paucibacillary leprosy, the odds ratio for leprosy classification is estimated imprecisely; it is reported with its confidence interval and interpreted with caution rather than being excluded. Scores from the PHQ-9 and SALSA were non-normally distributed (Shapiro-Wilk p < 0.001), so group comparisons of mean scores used the Mann-Whitney U test for two groups and the Kruskal-Wallis test for three.

### Ethical considerations

Ethical approval for the study was obtained from the Institutional Review Board (IRB) of Shaheed Suhrawardy Medical College. The ERC number is No-ShSMC/Ethical/2024/12. Written informed consent was secured from all participants prior to their enrollment. Confidentiality and anonymity were maintained throughout the study, and participants were assured of their right to withdraw at any time without consequences.

## Results

The study analyzed the socio-demographic, physical, mental and social profiles of 176 patients with leprosy, revealing a higher prevalence of leprosy among males (73.9%) compared to females (26.1%) which can be seen in Table 1. The mean age of participants was 54.14 ± 14.98 years. Majority of the patients were males (73.9%), married (79%) and farmers (23.9%).

**Table 1:** Sociodemographic characteristics of people affected by leprosy.

Variable	Female n= 46 (%)	Male n= 130 (%)	Total n= 176 (%)
Age			
<20	0	2 (1.5%)	2 (1.1%)
20-39	6 (13.0%)	24 (18.5%)	30 (17.0%)
40-60	26 (56.5%)	66 (50.8%)	92 (52.3%)
>60	14(30.4%)	38 (29.2%)	52 (29.5%)
Mean±SD	54.14 ± 15.21		
Marital Status			
Divorced	4 (8.7%)	3 (2.3%)	7 (4.0%)
Married	33 (71.7%)	106 (81.5%)	139 (79.0%)
Unmarried	6 (13.0%)	7 (5.4%)	13 (7.4%)
Widowed	3 (6.5%)	14 (10.8%)	17 (9.7%)
Religion			
Islam	35 (76.1%)	120 (92.3%)	155 (88.1%)
Hinduism	11 (23.9%)	9 (6.9%)	20 (11.4%)
Christianity	(0.0%)	1 (0.8%)	1 (0.6%)
Occupation			
Business	(0.0%)	24 (18.5%)	24 (13.6%)
Employed	2 (4.3%)	26 (20.0%)	28 (15.9%)
Farmer	(0.0%)	42 (32.3%)	42 (23.9%)
Homemaker	39 (84.8%)	1 (0.8%)	40 (22.7%)
Retired	(0.0%)	16 (12.3%)	16 (9.1%)
Student	(0.0%)	3 (2.3%)	3 (1.7%)
Unemployed	3 (6.5%)	10 (7.7%)	13 (7.4%)
Others	2 (4.3%)	8 (6.2%)	10 (5.7%)
Family Monthly Income in Bangladeshi Taka (BDT)			
<10000	9 (19.6%)	18 (13.8%)	27 (15.3%)
10000-49999	37 (80.4%)	109 (83.8%)	146 (83.0%)
>50000	(0.0%)	3 (2.3%)	3 (1.7%)
Mean±SD	16238.64 ± 16254.14		
Residence			
Urban	12 (26.1%)	32 (24.6%)	44 (25.0%)
Sub Urban	3 (6.5%)	10 (7.7%)	13 (7.4%)
Rural	31 (67.4%)	88 (67.7%)	119 (67.6%)

*Note. BDT, Bangladeshi taka; SD, standard deviation. Percentages are column percentages within each sex, and within the total sample for the final column.*

Table 1 shows that the largest age group was 40–60 years, comprising 56.5% of female and 50.8% of male participants, while 13.0% of female and 18.5% of male participants were aged 20–39 years. Among the female patients, 8.7% got divorced, compared to 2.3% of the male patients. For occupational status, 84.8% of the female patients are homemakers, and 32.3% of the male patients are farmers. Almost the same percentage (67.7%) of the male patients are from rural areas, compared to 67.4% of the female patients who are from rural areas.

**Fig 1:**
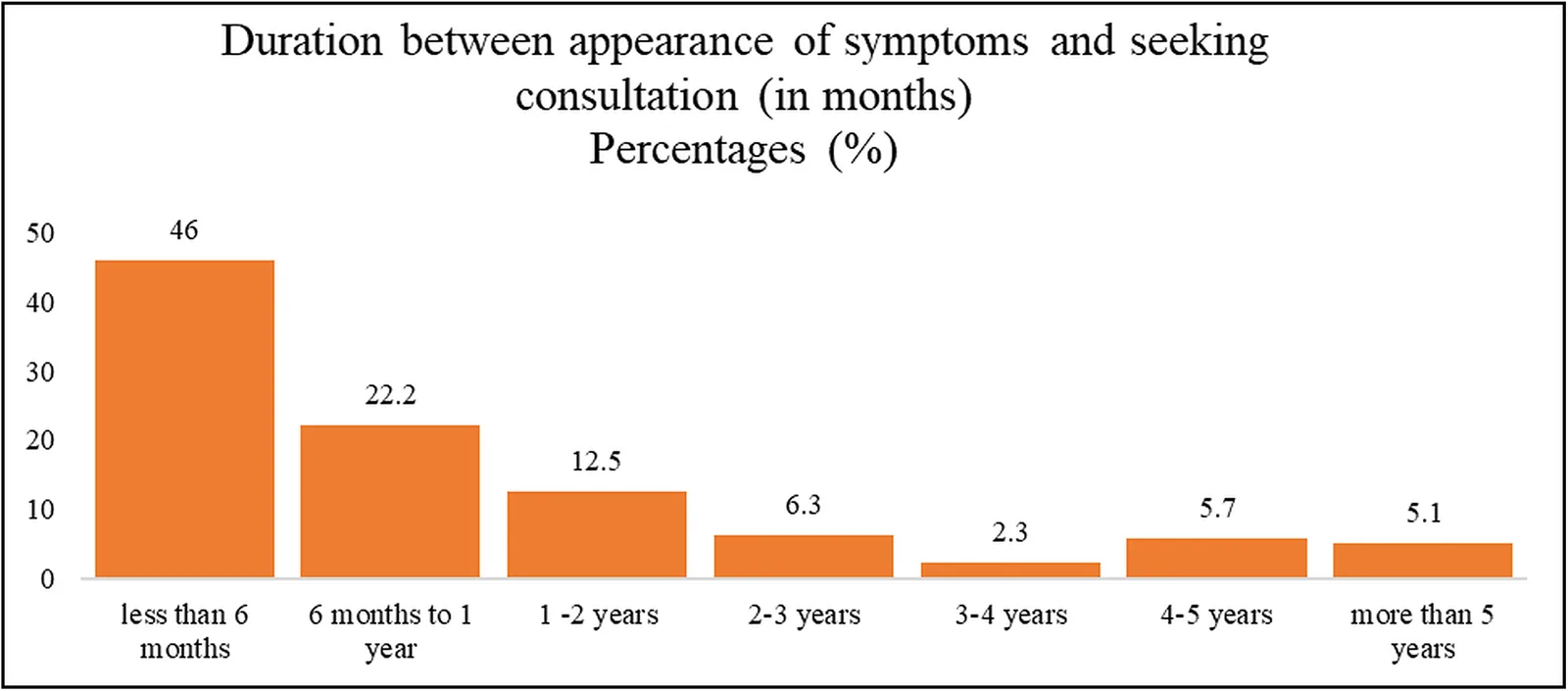
Duration between appearance of symptoms and seeking consultation for leprosy.

The relationship between appearance of symptoms and seeking consultation for leprosy is shown in Fig 1. Majority (46%) of patients sought consultation within the first six months. 22.2% of patients with leprosy sought consultation within 6 months to 1 year. It is noticeable that the majority of patients tend to seek consultation within one year.

**Fig 2:**
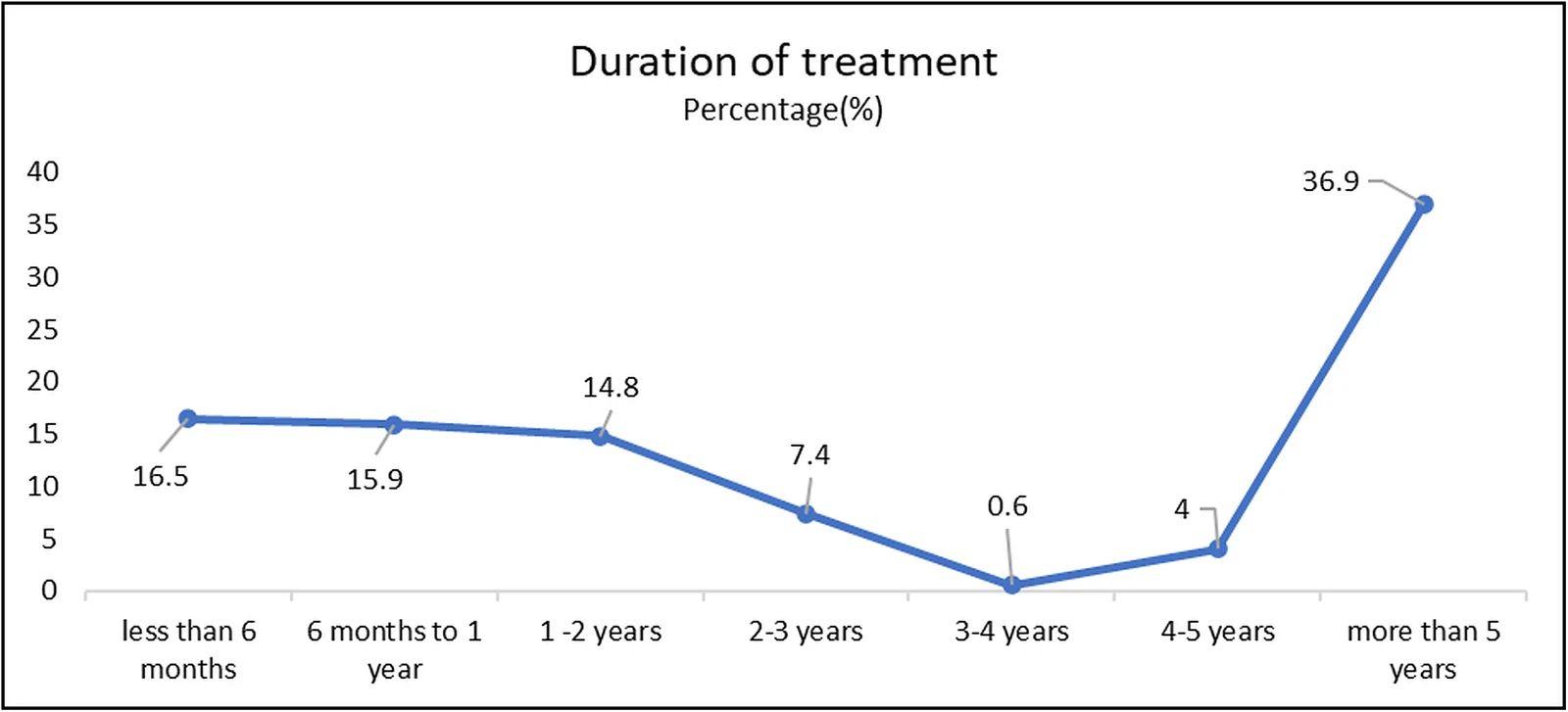
Duration of the treatment for the patients.

The duration of treatment for patients with leprosy in percentages is shown in Fig 2. Initially, approximately 16.5 percent of patients received treatment within six months. Then the line slopes downward till 4 years, indicating that the percentage of people seeking treatment decreases after 6 months till 4 years. It is observed that in the “more than 5 years” category, 36.9% of patients with leprosy continue their treatment.

Majority of the patients had multibacillary type of leprosy (94%) and suffered from Grade 2 impairments (72.7%). The most frequent impairments were nerve abscess (89.2%, 157/176), secondary ulcer (72.2%, 127/176), claw hands (54.5%, 96/176), foot drop (46.6%, 82/176), primary ulcer (44.3%, 78/176) and wrist drop (18.2%, 32/176). Ocular findings comprised pain or discomfort (65.3%, 115/176), dimness of vision (65.3%, 115/176), photophobia (28.4%, 50/176), keratitis (10.8%, 19/176) and lagophthalmos (5.1%, 9/176). Overall impairment grade did not differ significantly by sex (p = 0.21), but several individual impairments did, as summarized in Table 2 and Table 3 below.

**Table 2:** Distribution of impairment grading by sex.

Grading	Female n= 46 (%)	Male n= 130 (%)	Total n= 176 (%)
Impairment Grading			
Grade 0	(0.0%)	6 (4.6%)	6 (3.4%)
Grade 1	9 (19.6%)	33 (25.4%)	42 (23.9%)
Grade 2	37 (80.4%)	91 (70.0%)	128 (72.7%)

*Note. Impairment grade did not differ significantly by sex (p = 0.211). The minimum expected cell count was 1.57, so Fisher’s exact test was also applied and gave the same conclusion. Collapsing Grade 2 against Grades 0–1 gave chi-square = 1.38, df = 1, p = 0.241 (Fisher’s exact p = 0.185; odds ratio 1.76, 95% CI 0.78–4.00).*

**Table 3:** Association between deformities and sex.

Deformities	Sex of the respondents		p-value (BH-adjusted)
**Male** **n= 130 (%)**	**Female** **n= 46 (%)**
Claw hands	65 (50.0%)	31 (67.39%)	0.042 (0.107)
Wrist drops	21 (16.15%)	11 (23.91%)	0.24 (0.33)
Foot drop	52 (40.0%)	30 (65.22%)	0.003 (0.021)
Nerve abscess	119 (91.54%)	38 (82.61%)	0.10 (0.16)
Ocular Complications			
Lagophthalmos	7 (5.38%)	2 (4.35%)	1.0 (1.0)
Keratitis	10 (7.69%)	9 (19.57%)	0.048 (0.107)
Pain or discomfort	88 (67.69%)	27 (58.70%)	0.27 (0.33)
Photophobia	43 (33.08%)	7 (15.22%)	0.021 (0.077)
Dimness of vision	84 (64.62%)	31 (67.39%)	0.73 (0.81)
Ulcer			
Primary ulcer	66 (50.77%)	12 (26.09%)	0.004 (0.021)
Secondary ulcer	89 (68.46%)	38 (82.61%)	0.07 (0.12)

*Note. Values are n (%) within each sex. p-values are from Pearson’s chi-square test except for nerve abscess, lagophthalmos and keratitis, where an expected cell count below five required Fisher’s exact test. Figures in parentheses are p-values adjusted for the eleven simultaneous comparisons by the Benjamini-Hochberg false discovery rate procedure; only foot drop and primary ulcer remain significant after adjustment. These comparisons are exploratory.*

Among participants who had each impairment, the proportion classified as Grade 2 was 93.8% for wrist drop, 90.2% for foot drop, 89.5% for keratitis, 88.9% for lagophthalmos, 88.2% for secondary ulcer, 86.5% for claw hands, 80.0% for photophobia, 73.9% for nerve abscess, 73.9% for dimness of vision, 70.4% for ocular pain or discomfort and 53.8% for primary ulcer. These are conditional proportions within each impairment group, not prevalences in the sample; the figures reported in the original submission were of this kind and have been relabeled accordingly.

We can see that 80.4% of the female and 70% of the male patients have a grade 2 deformity from the Table 2. No female patients with Grade 0 are found in the limb deformity section, while 4.6% of the male patients have Grade 0 deformities.

The association between the respondents’ sex and various deformities is shown in Table 3. Females exhibited higher frequencies of claw hands (67.39%), foot drop (65.22%), and keratitis (19.57%), while males showed higher rates of lagophthalmos (5.38%), photophobia (33.08%), pain or discomfort (67.69%), and primary ulcers (50.77%). Although males generally experienced more nerve abscesses (91.54%), the difference was not statistically significant. Before adjustment for multiple comparisons, differences reached nominal significance for claw hands (p = 0.042), foot drop (p = 0.003), keratitis (p = 0.048), photophobia (p = 0.021) and primary ulcers (p = 0.004); after Benjamini-Hochberg correction across the eleven comparisons, only foot drop (adjusted p = 0.021) and primary ulcer (adjusted p = 0.021) remained significant. These exploratory findings suggest that the pattern of physical impairments differs between male and female patients with leprosy.

Moreover, the association between type of leprosy and disability grade is statistically significant (Fisher’s exact test, p < 0.001; the chi-square approximation is not appropriate here because two of six cells have expected counts below five) which can be seen from Table 4 below. According to the SALSA scale, 58% of patients had no significant limitations, 41.5% had mild limitations, and 0.6% had moderate limitations.

**Table 4:** Association Between Types of Leprosy and Disability Grades.

Types of leprosy	Grade 0	Grade 1	Grade 2	Total	*p*-value
Multibacillary	2 (33.3%)	37 (88.1%)	126 (98.4%)	165 (93.8%)	<0.001
Paucibacillary	4 (66.7%)	5 (11.9%)	2 (1.6%)	11 (6.3%)	

*Note. Values are n (%) within each impairment grade (column percentages). Two of the six cells had expected counts below five, so Fisher’s exact test was used (p < 0.001); the chi-square approximation is not appropriate here. Read by row, Grade 2 impairment occurred in 76.4% (126/165) of participants with multibacillary and 18.2% (2/11) of those with paucibacillary leprosy.*

Among participants with Grade 2 impairment, 98.4% had multibacillary leprosy, as did 88.1% of those with Grade 1 impairment which is shown in Table 4. By contrast, two-thirds (66.7%) of participants with Grade 0 impairment had paucibacillary disease. Only 2 of the 11 participants with paucibacillary leprosy (18.2%) had Grade 2 impairment, compared with 126 of 165 (76.4%) of those with multibacillary disease. The p-value (<.001) suggests that the association between types of leprosy and disability grades is statistically significant.

In case of mental disability assessment, 32.4% experienced minimal depression, 38.6% had mild depression, 21.0% had moderate depression, 7.4% had moderately severe depression, and 0.6% suffered from severe depression.

**Table 5:** Distribution of patients with leprosy according to depression severity.

Depression Severity	Frequency (%)
Minimal Depression	57 (32.4%)
Mild Depression	68 (38.6%)
Moderate Depression	37 (21.0%)
Moderately severe Depression	13 (7.4%)
Severe Depression	1 (0.6%)
Total	176 (100.0%)

*Note. PHQ-9 severity categories: minimal 0–4, mild 5–9, moderate 10–14, moderately severe 15–19, severe 20–27. A score of 5 or above indicates screening positive for at least mild depressive symptoms (67.6%); a score of 10 or above indicates moderate or worse symptoms (29.0%).* The depression severity among the patients, the majority of 38.6% had mild depression and 32.4% had minimal depression. In this study, only 0.6% of patients had severe depression shown in Table 5.

**Fig 3:**
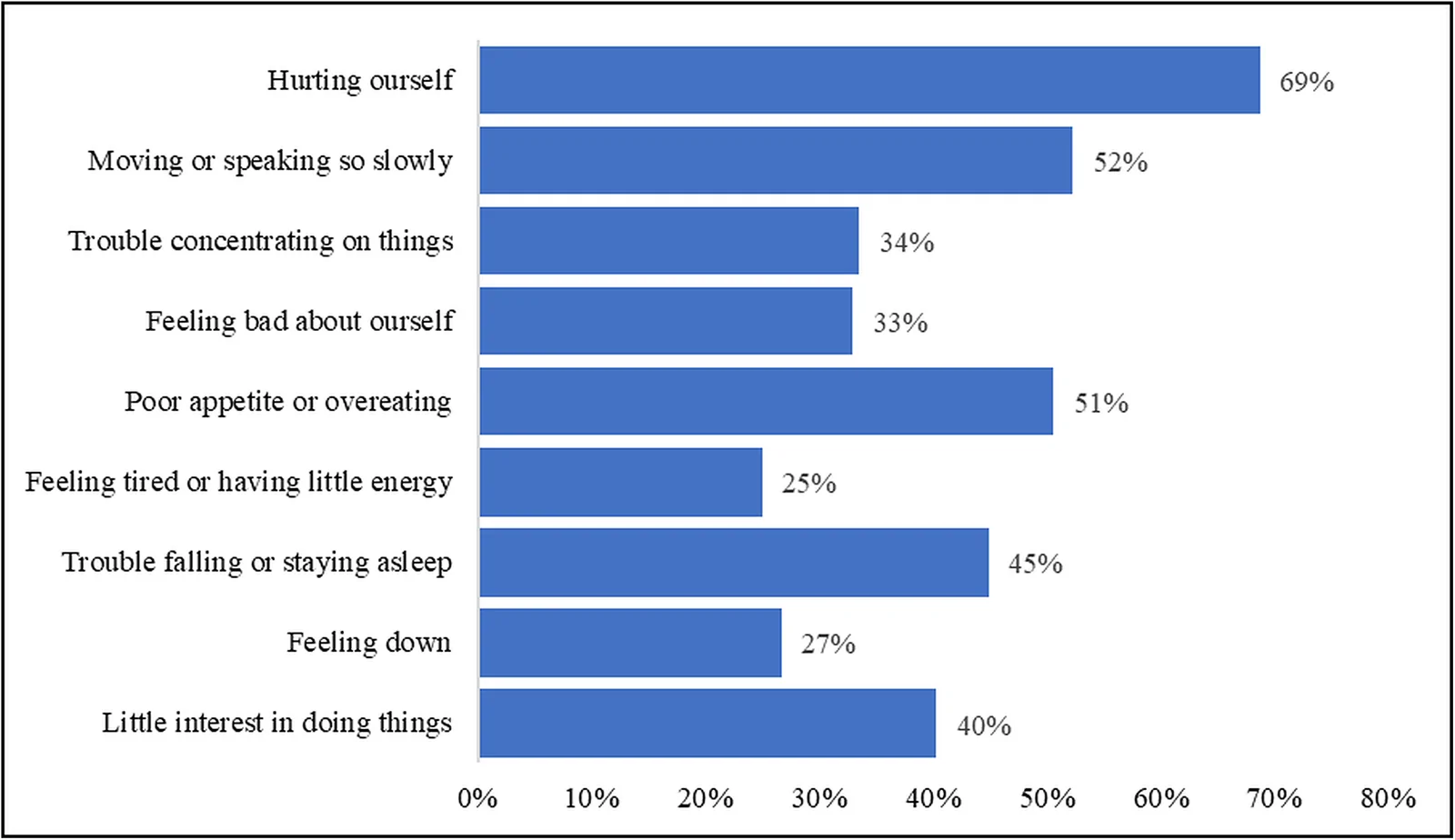
Mental Disability Assessment of Patients with Leprosy. (According to Patient Health Questionnaire-9).

The proportion of participants endorsing each PHQ-9 item, an item being counted as endorsed where the participant reported the symptom on at least several days in the preceding two weeks is shown in Fig 3. Fatigue or low energy was the most frequently endorsed symptom (75.0%), followed by depressed mood (73.3%), feelings of worthlessness or failure (67.0%), poor concentration (66.5%), anhedonia (59.7%) and disturbed sleep (55.1%). Poor appetite or overeating was endorsed by 49.4% and psychomotor slowing or agitation by 47.7%, while 31.2% reported any thoughts of self-harm or of being better off dead.

In case of social discrimination, 30.1% did not attend school (2.4% forced to leave), 8% were not married (28.6% unable to marry, 8.6% separated), 24.1% were forced to leave jobs, 20.4% had promotions affected, 12.8% were refused employment, 10.3% were restricted in jobs, 8% were refused public transport, 16% were refused entry to places of worship, 14.1% were denied access to public places, 2.3% were refused medical care, all of which is summarized in Table 6 below.

**Table 6:** Discrimination questionnaire for patients with leprosy.

Discriminations	Yes	No
1. Have you ever been at school?	123 (69.9%)	53 (30.1%)
-If NO Have you ever not been admitted to school or other education because being affected by Leprosy?	0 (0%)	53 (100%)
-If YES, have you ever been forced to leave or removed from school/other education because being affected by Leprosy?	3 (2.4%)	120 (97.6%)
2. Have you ever been married?	162 (92%)	14 (8%)
-If NO Have you ever been not able to marry because of being affected by Leprosy?	4 (28.6%)	10 (71.4%)
-If YES, have you ever been left or separated/divorced by your spouse because being affected by Leprosy?	14 (8.6%)	148 (91.4%)
3. Have you ever been working?	137 (77.8%)	39 (22.2%)
-If No, have you ever been refused employment because being affected by Leprosy?	5 (12.8%)	34 (87.2%)
-If No, have you ever been restricted or banned in your employment for certain areas, e.g., as cook or domestic helper because being affected by Leprosy?	4 (10.3%)	35 (89.7%)
-If yes, have you ever been forced to leave or removed from a job because being affected by Leprosy	33 (24.1%)	104 (75.9%)
-If yes, have your promotion ever been negatively affected because being affected by Leprosy?	28 (20.4%)	109 (79.6%)
4. Did you ever use public transportation?	163 (92.6%)	13 (7.4%)
-If YES, have you ever been refused travel by public transport because being affected by Leprosy?	13 (8%)	150 (92%)
5. Have you ever been at places of worship (temple, church, mosque or other)?	156 (88.6%)	20 (11.4%)
-If YES, have you ever been refused admission into a temple, church, mosque or other places of worship because being affected by Leprosy?	25 (16%)	131 (84%)
6. Have you ever been to public places like shops or restaurants?	149 (84.7%)	27 (15.3%)
-If YES, have you ever been refused admission into a restaurant, hotel, shops etc because of being affected by Leprosy?	21 (14.1%)	128 (85.9%)
7. Have you ever been seeking medical care in a hospital or a health center or at a doctor’s praxis?	131 (74.4%)	45 (25.6%)
-If YES, have you ever been refused medical care or hospital/clinic admission because of being affected by Leprosy?	3 (2.3%)	128 (97.7%)
8. Are you old enough to vote or be voted?	162 (92%)	14 (8%)
-If YES, have you ever been banned from standing for elections or refused to vote because of being affected by Leprosy?	1 (0.6%)	161 (99.4%)
9. Have you ever experienced other discriminatory situations?	79 (44.9%)	97 (55.1%)

We can see that a small percentage (2.4%) of patients with leprosy had to drop out of school due to their leprosy, many patients (69.9%) attended school in Table 6. The majority of participants (92%) are married, with 8.6% having separated because of the disease. According to employment statistics, 77.8% of people have received employment, 12.8% have not been employed, and 24.1% have been forced to quit because of leprosy. Although 92.6 percent of people used public transportation, 8% of people were not using it due to their disease. Public spaces (14.1%) and places of religion (16%) reported discrimination. Rejections for medical care were rare (2.3%), and 0.6% of the patients reported refusing to attend the voting.

Activity limitation, measured with the SALSA scale, was absent or minimal in most participants: 102 (58.0%) scored in the no-significant-limitation range, 73 (41.5%) reported mild limitation and 1 (0.6%) moderate limitation, with none in the severe or extreme range (Table 7). Limitation increased with impairment grade, from a mean SALSA score of 22.33 in Grade 0 to 23.26 in Grade 1 and 25.90 in Grade 2 (Kruskal-Wallis p < 0.001), and was greater among participants with multibacillary than paucibacillary disease (25.33 versus 22.45; Mann-Whitney p = 0.025).

**Table 7:** Distribution of activity limitation measured by the SALSA scale.

SALSA category	Score range	n (%)
No significant limitation	10-24	102 (58.0%)
Mild limitation	25-39	73 (41.5%)
Moderate limitation	40-49	1 (0.6%)
Severe limitation	50-59	0 (0.0%)
Extreme limitation	60-80	0 (0.0%)
Total		176 (100.0%)

*Note. SALSA, Screening of Activity Limitation and Safety Awareness. Scores range from 10 to 80, with higher scores indicating greater limitation. Mean SALSA score was 25.15 (SD 4.57).*

PHQ-9 severity and mean PHQ-9 score against disability grade, leprosy classification, treatment duration, experience of discrimination and sex presents in Table 8. Depressive symptoms increased with impairment grade: no participant with Grade 0 impairment screened positive, compared with 57.1% of those with Grade 1 and 74.2% of those with Grade 2 (chi-square = 18.59, df = 4, p < 0.001), with mean scores of 1.67, 6.74 and 7.48 respectively (Kruskal-Wallis p = 0.004). Mean scores were higher in multibacillary than paucibacillary disease (7.35 versus 3.45; Mann-Whitney p = 0.006), although the severity distribution did not differ significantly (p = 0.063). Scores rose with treatment duration (5.66, 6.97 and 7.80 for 6 months or less, 6 months to 5 years, and more than 5 years) but not significantly (Kruskal-Wallis p = 0.103). Participants reporting any experience of discrimination scored higher than those who did not (7.75 versus 6.25; Mann-Whitney p = 0.023). Women scored substantially higher than men (8.72 versus 6.53; Mann-Whitney p = 0.002), and 43.5% of women compared with 23.8% of men scored 10 or above, despite showing no significant difference in overall impairment grade.

**Table 8:** PHQ-9 severity and mean score by disability grade, leprosy classification, treatment duration, experience of discrimination and sex.

Characteristic	n	Minimal (0–4)	Mild (5–9)	Moderate or worse (≥10)	Mean±SD	p (distribution)	p (mean score)
**Disability grade**						<0.001	0.004
Grade 0	6	6 (100.0%)	0 (0.0%)	0 (0.0%)	1.67 ± 1.86		
Grade 1	42	18 (42.9%)	11 (26.2%)	13 (31.0%)	6.74 ± 5.78		
Grade 2	128	33 (25.8%)	57 (44.5%)	38 (29.7%)	7.48 ± 4.28		
**Leprosy classification**						0.063	0.006
Multibacillary	165	50 (30.3%)	65 (39.4%)	50 (30.3%)	7.35 ± 4.65		
Paucibacillary	11	7 (63.6%)	3 (27.3%)	1 (9.1%)	3.45 ± 4.55		
**Duration of treatment**						0.166	0.103
6 months or less	29	12 (41.4%)	13 (44.8%)	4 (13.8%)	5.66 ± 4.20		
More than 6 months to 5 years	75	24 (32.0%)	31 (41.3%)	20 (26.7%)	6.97 ± 4.97		
More than 5 years	65	19 (29.2%)	21 (32.3%)	25 (38.5%)	7.80 ± 4.41		
**Any experience of discrimination**						0.115	0.023
No	76	31 (40.8%)	26 (34.2%)	19 (25.0%)	6.25 ± 4.73		
Yes	100	26 (26.0%)	42 (42.0%)	32 (32.0%)	7.75 ± 4.64		
**Sex**						0.012	0.002
Male	130	49 (37.7%)	50 (38.5%)	31 (23.8%)	6.53 ± 4.90		
Female	46	8 (17.4%)	18 (39.1%)	20 (43.5%)	8.72 ± 3.80		

*Note. PHQ-9, Patient Health Questionnaire-9. Values are n (%) within each row category. Distribution p-values are from Pearson’s chi-square test across the three collapsed severity categories; the five-category tables were not tested because expected counts fell below one. Mean-score p-values are from the Mann-Whitney U test (two groups) or the Kruskal-Wallis test (three groups), scores being non-normally distributed (Shapiro-Wilk p < 0.001). Treatment duration was missing for seven participants (n = 169).*

In the multivariable model for Grade 2 impairment (Table 9; n = 169, seven participants excluded for missing treatment duration), multibacillary classification was the strongest independent predictor (adjusted OR 15.70, 95% CI 1.94–126.75, p = 0.010), and each additional year of treatment was associated with slightly raised odds (adjusted OR 1.08, 95% CI 1.00–1.16, p = 0.043). Rural residence (adjusted OR 2.18, 95% CI 0.95–5.00, p = 0.067) and each additional month of delay between symptom onset and first consultation (adjusted OR 1.03, 95% CI 1.00–1.05, p = 0.061) approached but did not reach significance, while age (p = 0.496) and female sex (p = 0.294) were not independently associated with Grade 2 impairment. The model discriminated moderately (area under the ROC curve 0.779) with adequate calibration (Hosmer-Lemeshow p = 0.50; McFadden pseudo-R-squared 0.203). The confidence interval for multibacillary classification is very wide because only 11 participants had paucibacillary disease, and this estimate should be interpreted with corresponding caution.

**Table 9:** Multivariable logistic regression for Grade 2 impairment and for moderate or worse depressive symptoms.

Predictor	Adjusted OR	95% CI	p-value
**Model 1: Grade 2 impairment (n = 169)**			
Age (per year)	1.01	0.98-1.04	0.496
Female sex (versus male)	1.65	0.65-4.24	0.294
Multibacillary (versus paucibacillary)	15.70	1.94-126.75	0.010
Delay, onset to first consultation (per month)	1.03	1.00-1.05	0.061
Duration of treatment (per year)	1.08	1.00-1.16	0.043
Rural residence (versus urban or suburban)	2.18	0.95-5.00	0.067
**Model 2: PHQ-9 score of 10 or above (n = 176)**			
Age (per year)	0.99	0.97-1.01	0.347
Female sex (versus male)	2.50	1.21-5.14	0.013
Impairment grade (per grade)	1.17	0.59-2.33	0.652
Any experience of discrimination (versus none)	1.41	0.70-2.82	0.332

*Note. OR, odds ratio; CI, confidence interval; PHQ-9, Patient Health Questionnaire-9. Model 1: McFadden pseudo R-squared 0.203, area under the ROC curve 0.779, Hosmer-Lemeshow p = 0.50; seven participants were excluded for missing treatment duration. Model 2: McFadden pseudo R-squared 0.039, area under the ROC curve 0.627. Only 11 participants had paucibacillary leprosy, which accounts for the wide confidence interval on that estimate in Model 1.*

In a second model with moderate or worse depressive symptoms (PHQ-9 score of 10 or above) as the outcome, female sex was the only independent predictor (adjusted OR 2.50, 95% CI 1.21–5.14, p = 0.013); impairment grade (adjusted OR 1.17 per grade, 95% CI 0.59–2.33, p = 0.652), any experience of discrimination (adjusted OR 1.41, 95% CI 0.70–2.82, p = 0.332) and age (adjusted OR 0.99, 95% CI 0.97–1.01, p = 0.347) were not.

## Discussion

The study “Assessment of disability of Leprosy in Bangladesh: A hospital-based study” provides a comprehensive analysis of the demographic, clinical, and psychological profiles of 176 patients with leprosy. The demographic data indicate a higher prevalence of leprosy among males (73.9%) compared to females (26.1%). The mean age of the participants was 54.14 years. This distribution of age correlates with study results conducted in India, where a greater prevalence of leprosy in older ages was observed [18]. A total of 79% of participants were married, 88.1% were Muslim, and there were large gender variations in occupation; 84.8% of the females are homemakers, and 32.3% of the males are farmers. This socio-economic context is critical to learn the weight of leprosy as Smith et al. (2019) have done, where socio-economic issues are found to be a major contributor to the prevalence of leprosy in Brazil [19].

Findings on impairment grade show that 72.7% of participants had Grade 2 impairment. The proportion was higher among women (80.4%) than men (70.0%), but this difference was not statistically significant (p = 0.21) and should not be interpreted as a sex difference in overall disability severity. These findings highlight the high disability burden on the patients of leprosy. Grade 2 impairments was considerably more common among participants with multibacillary leprosy (76.4%, 126/165) than among those with paucibacillary leprosy (18.2%, 2/11; Fisher’s exact p < 0.001). Expressed the other way round, 98.4% of all participants with Grade 2 impairment had multibacillary disease. This is consistent with other studies which reported the same trends with the high rates of severe disabilities in multibacillary patients in India and Brazil respectively. [20,21]. The critical aspects of the disease in India and Brazil are a reflection of the broadened bacterial burden and chronicity of the disease, and the need to target diagnoses and interventions, respectively.

Nerve abscess was the most frequent impairment (89.2%), followed by secondary ulcer (72.2%), claw hands (54.5%) and foot drop (46.6%); wrist drop was recorded in 18.2%. Claw hands were more frequent among women than men (67.4% versus 50.0%, unadjusted p = 0.042), although this difference did not persist after correction for multiple comparisons (Benjamini-Hochberg adjusted p = 0.107). Because the study is cross-sectional, these impairments are described as co-occurring rather than causally related. These results conform to a study that indicated similar patterns of deformities in India [22]. Ocular findings were also common: dimness of vision affected 65.3% of participants and ocular pain or discomfort 65.3%, while lagophthalmos was comparatively uncommon at 5.1%. These eye problems have a serious effect on the quality of life, just as was reported by the research conducted on leprosy in China [23,24].

This paper explores gender differences in leprosy-related deformities in Bangladesh, with foot drop more prevalent among women (65.2% versus 40.0%; unadjusted p = 0.003, Benjamini-Hochberg adjusted p = 0.021) and primary ulcer more prevalent among men (50.8% versus 26.1%; unadjusted p = 0.004, adjusted p = 0.021), the former being consistent with the study by Croft et al. (1999) that showed that women had delayed access to healthcare [25]. Nerve abscess was somewhat more frequent among men (91.5% versus 82.6%) and photophobia markedly so (33.1% versus 15.2%), but neither difference persisted after adjustment (adjusted p = 0.162 and 0.077 respectively) [26,27]. Dimness of vision was almost identical in both sexes (64.6% of men, 67.4% of women) and lagophthalmos uncommon in both (5.4% versus 4.3%; Fisher’s exact p = 1.0), while differences in claw hands, wrist drop, keratitis, ocular pain and secondary ulcer did not persist after adjustment [28–31]. These results highlight the significance of gender-sensitive leprosy care to respond to certain healthcare obstacles faced by women. All eleven sex comparisons in Table 3 are exploratory and are interpreted as such.

The research uses the Screening of activity limitation and safety awareness (SALSA) scale to determine functional limitations. Across the sample, 58.0% reported no significant activity limitation, 41.5% mild limitation and 0.6% moderate limitation. Limitation was more common among participants with multibacillary disease, of whom 43.0% reported mild and 0.6% moderate limitation, than among those with paucibacillary disease, of whom 18.2% reported mild limitation and none moderate (mean SALSA score 25.33 versus 22.45; Mann-Whitney p = 0.025). Limitation also increased with impairment grade: mild or worse limitation was reported by 16.7% of participants with Grade 0, 23.8% with Grade 1 and 49.2% with Grade 2 impairment (mean score 22.33, 23.26 and 25.90 respectively; Kruskal-Wallis p < 0.001). Those findings are in agreement with other studies that also reported increased limitations in the daily activities and safety awareness among the multibacillary patients [32,33]. The fact that multibacillary patients have high limitations in their everyday life as well as in safety awareness supports the idea that comprehensive care strategies are necessary to address the needs of these patients.

In the mental health assessment, 38.6% of participants had mild depressive symptoms, 21.0% moderate, 7.4% moderately severe and 0.6% severe, with 32.4% in the minimal range. At item level, the most frequently endorsed symptoms were fatigue or low energy (75.0%), depressed mood (73.3%), feelings of worthlessness or failure (67.0%) and poor concentration (66.5%); poor appetite or overeating was endorsed by 49.4% and psychomotor change by 47.7%, while 31.2% reported any thoughts of self-harm or of being better off dead. The results are in line with a comparable study in Bangladesh, which identified a substantial mental health burden and stigma among people affected by leprosy [34]. The overall burden of depressive symptoms observed here is consistent with the psychological burden and stigma reported among people affected by leprosy in Indonesia and the Philippines [35].

The results of the study are consistent with earlier studies that have shown that patients with leprosy have high levels of deformities and disabilities. These findings are in line with previous studies in different countries with leprosy that have reported that there is a consistent prevalence of deformities as a result of the prolonged duration of disease that have offered significant proof that leprosy and its complications are difficult to control [36,37]. In some studies, research on the reduction of these disabilities has been highlighted in order to have a leprosy free world. These findings are in line with studies conducted across different countries that are endemic to leprosy in that there is a persistent prevalence of deformities due to the extended duration of the disease.

On the side of the patient, the lack of awareness and knowledge are the significant factors associated with the postponement of the early detection of the disease [17,38]. In the regions with a high incidence of leprosy, many patients are not aware of the symptoms, mode of transmission, and treatment of the disease. Cultural misbeliefs related to leprosy (that it is a curse or a consequence of immoral actions, etc.) are also the causes that delay medical consultations and lead to more severe disabilities and an endless cycle of stigma and isolation [38]. Social stigma and isolation also contribute to the further worsening of the situation. Leprosy is stigmatized so that patients are often rejected by their families and communities not only impacting their mental health but also making them unwilling to take prompt treatment [39]. Social stigma and isolation further exacerbate the situation. Patients often face rejection from their families and communities due to the stigma associated with leprosy, which not only affects their mental health but also discourages them from seeking early treatment. In other situations, the patients can isolate themselves to prevent social ostracization, which further postpones the diagnosis and treatment [40].

One other economic barrier is that of early detection. Patients with leprosy belong to low socio-economic groups and cannot afford medical services, and therefore many of them do not receive medical services early, contributing to the condition [41,42]. The fear of job loss or reduced earning capacity prevents many from seeking early diagnosis and treatment, exacerbating their condition.

As seen through the eyes of the health professionals, lack of adequate training and awareness is a key obstacle to efficient early detection [43]. Health workers and the local level traditional health-related service providers in endemic areas tend to lack the necessary training to detect leprosy at its initial stages [44]. Misdiagnosis or slow diagnosing by the healthcare provider can be a significant issue that affects the results of the patient [45]. Comprehensive training and awareness of the healthcare providers towards the patients with leprosy is crucial in allowing the service provider to detect the disease at an earlier stage [46]. The attitude and perception that clinicians might have stigmatizing attitudes towards leprosy, which influence their attitude towards patients and motivate them to seek assistance or follow the treatment. This gap can be filled by community health programs which involve local health service providers as part of formal health systems to facilitate early-stage detection [39].

Early detection is crucial, particularly in rural and endemic regions, and requires training healthcare providers to recognize the early signs of leprosy and to initiate treatment promptly. Moreover, multibacillary cases are to receive priority because they are more likely to give severe disabilities and bacterial load. Full-care plans ought to be formulated which involve medical treatment as well as rehabilitation to patients whose impairment is Grade 2. In addition, assistive devices, such as prosthesis and physiotherapy should be provided to patients with impairments like foot drop, wrist drop, and claw hands. Moreover, socio-economic support should also be given to affected individuals through microfinance schemes, vocational training, and employment opportunities and mental health services should be improved by providing counseling services and peer support groups to help patients cope with stigma and social isolation and depression and anxiety. Additionally, public awareness campaigns should be conducted to reduce the stigma associated with leprosy, promoting understanding and acceptance within communities and patients and their families should be educated about self-care practices and the importance of adherence to treatment regimens.

The unwillingness of patients to seek help with leprosy during its early stages is one of the major reasons for late detection. And apart from dermatology, doctors of other fields are not even able to identify the diseases and provide the patients with medicinal support. Therefore, in hotspot regions where leprosy is very prevalent such as Nilphamari, Joypurhat, Panchagarh, Dinajpur, Thakurgaon and Rangpur from North-western region; tea gardens of Moulvibazar in Sylhet; Jalchatra in Mymensingh; Chandroghona, Kaptai; and Dhaka as the central hub as it is the capital of the country, extensive training should be provided to the doctors so that early detection of the disease is possible.

### Strengths and limitations

The strength of the study was in its holistic presentation as both clinical measurements (WHO disability grading) are incorporated with psychosocial and functional measurements (PHQ-9 and SALSA scale). Nevertheless, the results should be deciphered with some limitations. The convenience sampling of five hospital centers has presented the risk of selection bias, in that, the results cannot be completely applicable to the whole population of patients with leprosy in Bangladesh. Notably, a hospital-based design is prone to over-sample patients with severe disease and Grade 2 impairments and could be missing undiagnosed and lighter cases in the community. Also, the gathered self-reported information on sensitive topics, such as stigma and mental health, despite the use of standardized instruments, poses a threat of either response bias or social desirability bias.

Two further limitations warrant emphasis. First, the achieved sample of 176 fell well short of the calculated target of 371, reducing precision and limiting power to detect associations of modest size; some of the non-significant findings reported here, including the comparison of impairment grade by sex, may reflect this rather than a true absence of association. Second, and more importantly for interpretation, all five participating sites are specialist leprosy referral centers. Patients with visible deformity, ulceration, ocular complications or reaction are considerably more likely to attend such centers than those with early or uncomplicated disease, who may be managed in primary care or may not present at all.

The observed figures of 72.7% Grade 2 impairment and 94.0% multibacillary disease should therefore be read as the burden among people affected by leprosy attending specialist centers in Bangladesh, and not as the prevalence in the national leprosy population or among newly detected cases, for which the reported Grade 2 disability rate is substantially lower. Convenience sampling compounds this, since participants were those present and willing during the data-collection period rather than a probability sample. The likely net effect is an upward bias in disability grade, in the multibacillary proportion, and plausibly in the psychosocial outcomes, since both depressive symptoms and experienced discrimination are associated with visible deformity. The internal associations we report are less vulnerable to this selection than the prevalence estimates, though not immune to it.

## Conclusion

The extensive hospital-based research on the situation with leprosy in Bangladesh demonstrates the high weight of the neglected tropical disease with its high levels of both severe disability and related psychosocial problems. Among patients attending these centers, the disease was more frequent in older males, and there were marked gender differences in occupation and socio-economic status. Overall impairment grade did not differ significantly by sex, although the distribution of individual impairments did. Importantly, patients with Grade 2 impairment constituted 72.7% of this facility-based sample, with the burden concentrated among those with multibacillary (MB) disease.

The outcomes of these findings highlight the fact that late diagnosis and treatment continue to be the main causes of distress and serious disability. In Bangladesh, intervention strategies should focus on ensuring that early detection is done by enhancing training of all healthcare providers on how to identify the first signs of leprosy to be able to greatly reduce the burden of leprosy. In addition, successful programs will have to involve a transition to comprehensive care, such as rehabilitation services and assistive devices to individuals with Grade 2 impairments, better mental health services and specific publicity campaigns to decrease social stigma.

## Supporting information

S1 File Leprosy dataset. (XLSX)
