# Correction: Assessment of disability of leprosy in Bangladesh: A hospital-based study

**DOI:** 10.1371/journal.pone.0361054

**Published:** 2026-10-16

**Authors:** Hafez Mohammad Nazmul Ahsan, Shaheera Rahman, Labida Islam, Monjur Rahman, Farhana Zaman, Rowshan Ara, Hossain Mohammed Omar Khayum, Saidur Rahman Mashreky

In the Study design subsection of the Methodology, there is an error in the second sentence. The correct sentence is: The study utilized a cross-sectional study design. The study was conducted at Filaria, Thalassemia and General Hospital, Savar; Institute of Leprosy Control Hospital, Mohakhali; Government Leprosy Hospital, and The Leprosy Mission International Bangladesh (TLMI-B); and Filaria Hospital & CDC, Saidpur.

The following information is missing from the Acknowledgement section: We would like to acknowledge The Leprosy Mission International Bangladesh (TLMI-B) for providing permission for data collection at the hospital during the study period.
